# An Integrative Analysis of *PIK3CA* Mutation, PTEN, and INPP4B Expression in Terms of Trastuzumab Efficacy in HER2-Positive Breast Cancer

**DOI:** 10.1371/journal.pone.0116054

**Published:** 2014-12-26

**Authors:** Aiko Sueta, Yutaka Yamamoto, Mutsuko Yamamoto-Ibusuki, Mitsuhiro Hayashi, Takashi Takeshita, Satoko Yamamoto, Hirotaka Iwase

**Affiliations:** 1 Department of Breast and Endocrine Surgery, Kumamoto University Graduate School of Medical Science, 1-1-1, Honjo, Chuo-ku, Kumamoto, 860-8556, Japan; 2 Department of Molecular-Targeting Therapy for Breast Cancer, Kumamoto University Hospital, 1-1-1, Honjo, Chuo-ku, Kumamoto, 860-8556, Japan; University of Torino, Italy

## Abstract

The phosphoinositide-3-kinase (PI3K) pathway is commonly deregulated in breast cancer through several mechanisms, including *PIK3CA* mutation and loss of phosphatase and tensin homolog (PTEN) and inositol polyphosphate 4-phosphatase-II (INPP4B). We aimed to evaluate the predictive relevance of these biomarkers to trastuzumab efficacy in HER2-positive disease. We evaluated the effect of trastuzumab in 43 breast cancer patients with HER2-overexpression who received neoadjuvant treatment. *PIK3CA* mutation was examined by direct sequencing and digital PCR assay, and *PIK3CA* copy number was assessed by digital PCR assay of pretreatment tissues. PTEN, pAkt, and INPP4B were assessed by immunohistochemistry. Direct sequencing detected mutant DNA in 21% of all patients, but the incidence increased to 49% using digital PCR. The pathological complete response (pCR) rate in patients with *PIK3CA* mutations was 29% compared with 67% for those without *PIK3CA* mutations (*P* = 0.093), when the mutation was defined as positive if the mutant proportion was more than 10% of total genetic content by digital PCR. Low PTEN expression was associated with less pCR compared to high expression (33% versus 72%, *P* = 0.034). There were no significant associations of *PIK3CA* copy number, pAKt, or INPP4B with trastuzumab efficacy. In multivariate analysis, activation of the PI3K pathway due to either *PIK3CA* mutation or low PTEN were related to poorer response to trastuzumab (OR of predictive pCR was 0.11, 95%CI; 0.03–0.48). In conclusion, activating the PI3K pathway is associated with low pCR to trastuzumab-based treatment in HER2-positive breast cancer. Combined analysis of *PIK3CA* mutation and PTEN expression may serve as critical indicators to identify patients unlikely to respond to trastuzumab.

## Introduction

Anti-human epidermal growth factor 2 (HER2) therapy has been approved as a standard practice for patients with HER2-positive breast cancer, leading to an improvement of patient outcome during the past decade [1], [2], [3]. Despite the considerable efficacy of trastuzumab therapy, some patients with metastatic breast cancer either do not respond to it or have a limited benefit [4], [5]. This resistance to trastuzumab is a major issue in clinical practice and the molecular basis of the resistance has not been completely elucidated.

The phosphoinositide-3-kinase (PI3K) pathway, which is a downstream target of most growth factor tyrosine kinase receptors (TKRs) including HER2 and insulin-like growth factor-1 receptor (IGF1R), contributes to cell proliferation, metabolism, autophagy, and cell survival and also confers resistance to trastuzumab [6], [7]. Aberrations of this pathway are extensively found in many human cancers in a variety of forms, including mutation or amplification of *PIK3CA* and loss of phosphatase and tensin homolog (PTEN) and inositol polyphosphate 4-phosphatase-II (INPP4B) [8], [9], [10].

Activating mutations in the *PIK3CA* gene, which encodes the p110α catalytic subunit of PI3K, are frequent in breast cancer as are mutations in the *p53* gene [11], [12]. The frequency of *PIK3CA* mutations in HER2-positive breast cancer has been reported as 22.7% to 39% [11], [12], [13]. Approximately 90% of these mutations are localized in 3 major hotspots concentrated in the helical (E542K and E545K) and kinase (H1047R) domains [14]. Loss of function of PTEN, a negative regulator of PI3K signaling, has been reported in 15%–65% of HER2-positive breast cancer [7], [8], [15], [16], [17], [18]. Likewise, the expression of a putative tumor suppressor, INPP4B, is frequently lost in breast cancer, and is reported to be associated with decreased patient survival [10].

To date, a number of *in vitro* studies have demonstrated the putative mechanism of resistance to trastuzumab therapy in terms of PI3K pathway activation [7], [19], [20], but clinical confirmations of this association are limited. Chandaelapaty et al. revealed that the incidence of PTEN loss and/or *PIK3CA* mutation in trastuzumab-refractory tumors was 71% compared with 44% in primary tumors from an untreated cohort [21]. This supports the findings that showed that, during cancer evolution, tumors accumulate molecular or genetic events to overcome exposure to the drug [22]. When investigating the molecular basis of treatment resistance, it is difficult to obtain the tumor tissues consistently after disease progression in a metastastic setting. However, neoadjuvant treatment offers an opportunity to explore the efficacy of therapy and allows us to predict *de novo* resistance using primary tissues. To our knowledge, studies of the association between PI3K-pathway activation and pathological complete response (pCR) to trastuzumab treatment have been limited.

For the detection of mutations, direct sequencing has been commonly used in clinical research. However, it can only detect mutant sequences constituting more than 20% of the total genetic content [23]. Digital PCR technology with an analytical sensitivity of 0.01–0.1%, represents an attractive approach for the detection of low-abundance mutations [24], and allows accurate quantitative measurement of mutant DNA. A recent study reported that digital PCR could detect additional mutations in primary tumor tissues not found by traditional Sanger sequencing [25], suggesting that previous evaluations of mutations in pretreatment tumor tissues might have underestimated the true frequency.

The objective of this study is to evaluate the predictive relevance of PI3K-pathway related biomarkers to trastuzumab efficacy in HER2-positive disease. In a neoadjuvant setting, we can identify *de novo* resistance to trastuzumab therapy, which requires alternative treatment such as inhibitors of the PI3K/AKT/mTOR pathway in early stage of the disease. Additionally, we aim to determine accurately the frequency of *PIK3CA* mutation using the novel technology of digital PCR.

## Results

Patient characteristics in this study are summarized in S2 Table. The mean age at diagnosis was 53 years (range 30–75). Forty-four percent had tumors of the luminal-HER2 subtype and 56% HER2-enrich. Most patients (81%) received an anthracycline- and taxan-containing regimen in combination with trastuzumab in the neoadjuvant treatment. Of the 43 patients, 26 (60%) achieved pCR.

### Frequency of *PIK3CA* mutation detected by direct sequencing and digital PCR assays

**Figure 1:**
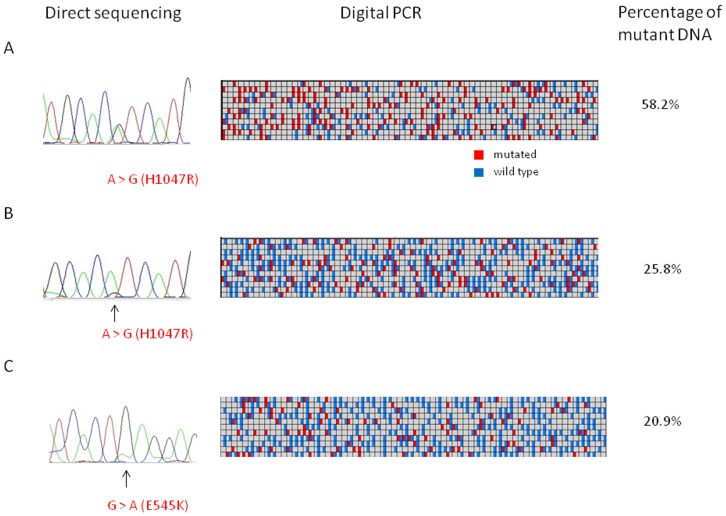
Representative image of *PIK3CA* exon 20 (A, B) and exon 9 mutations (C) using direct sequencing and digital PCR. For the digital PCR assay, signals from wild-type-specific probe are detected as blue, and signals from mutant-type are detected as red. A; mutation is detectable by direct sequencing and harbors more than 50% of mutant DNA using digital PCR. B, C; detection of mutation is difficult by direct sequencing alone, harbors around 20% of mutant DNA by digital PCR.

We first investigated *PIK3CA* mutations in the pretreatment tissues using direct sequencing, which confirmed 7 (16%) mutations of exon 20 and 3 (7%) of exon 9. Subsequently, the tissues were subjected to digital PCR assays, in which the fractional abundance of mutant DNA ranged from 0.1% to 58.2% (Table 1). The corresponding frequencies of *PIK3CA* mutations were 35% of exon 20 and 28% of exon 9. Fig. 1 shows representative images of the detection of mutant *PIK3CA* DNA by both techniques. The first illustrated case (Fig. 1A), in which an apparent mutation was detected by direct sequencing, showed more than 50% mutant DNA in a background of wild-type DNA by digital PCR analysis. In contrast, we found some cases in which it was difficult to differentiate mutant DNA from artifact by direct sequencing (Fig. 1B and C). In these cases, we could identify the mutation clearly using digital PCR (25.8% and 20.9%). Mutations detected by both assays are shown in Table 1. In total, direct sequencing detected mutant DNA in 21% of patients, but the incidence increased to 49% using digital PCR. Most mutations detected by direct sequencing were also identified by digital PCR, and the proportion of mutant DNA was more than 10% in all cases except numbers 15 and 41 (Table 1). In 12 (28%) samples, *PIK3CA* mutation was detected only using the digital PCR, as the mutant proportion was around 1% or less.

**Table 1:** Detection of *PIK3CA* mutation (E542K, E545K, H1047R) by DNA sequencing and digital PCR analysis (only among the patients whose mutations were detected).

Patient ID	Mutation	
	Direct sequencing Digital PCR analysis (mutant percentage)	
31	H1047R, E542K	H1047R (58.2%), E542K (41.0%)
10	H1047R	H1047R (28.9%)
40	H1047R	H1047R (25.8%), E542K (0.3%), E545K (0.2%)
18	H1047R	H1047R (19.2%)
37	H1047R	H1047R (16.7%), E542K (0.1%)
15	H1047R	H1047R (6.8%)
41	H1047R	E542K (0.2%)
27	E542K	E542K (24.6%)
8	E545K	E545K (20.9%)
23	Wild-type	H1047R (7.7%)
34	Wild-type	H1047R (5.1%), E545K (0.9%)
3	Wild-type	H1047R (1.5%)
16	Wild-type	H1047R (0.8%)
36	Wild-type	H1047R (0.7%)
1	Wild-type	H1047R (0.6%)
7	Wild-type	H1047R (0.6%)
12	Wild-type	H1047R (0.5%), E545K (0.3%)
26	Wild-type	H1047R (0.4%), E542K (0.1%)
13	Wild-type	E542K (0.4%)
39	Wild-type	E545K (0.3%)
28	Wild-type	E542K (0.1%), E545K (0.1%)
Frequency^a^	21%	49%

a Frequency of the patient with mutated DNA.

### Associations of *PIK3CA* mutation, gene copy number with efficacy of trastuzumab therapy

**Figure 2:**
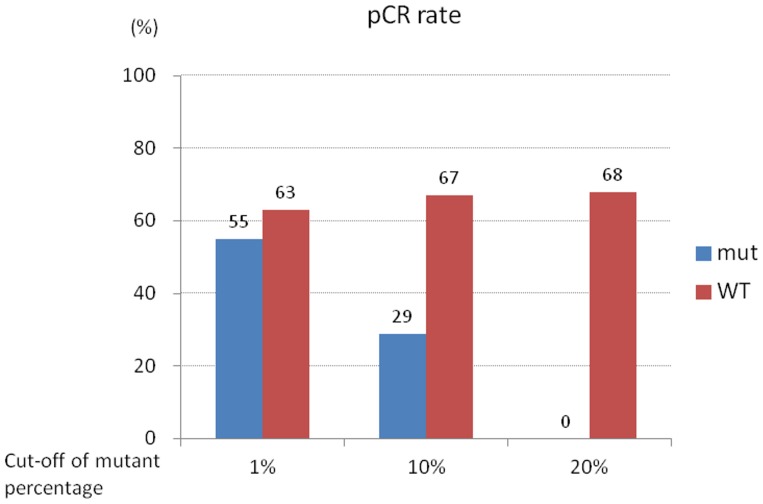
Correlation of proportion of *PIK3CA* mutation (defined by digital PCR) with the pCR rate. Mut; mutation, WT; wild type.

We next evaluated whether *PIK3CA* mutations influence the efficacy of trastuzumab therapy. As the significance of low-abundance mutation, especially those less than 1%, has been unclear, we set out to determine an appropriate cutoff point for the proportion of mutant DNA. Using a cutoff of 10%, the pCR rate in patients positive for *PIK3CA* mutations was 29% compared with 67% for those below the cutoff (*P* = 0.093, Fig. 2, Table 2). This effect was more evident when the cutoff point was 20%, with none of the mutation-positive patients achieving the pCR (Fig. 2). The location of the mutation in the *PIK3CA* gene (exon 9 vs. exon 20) made no significant difference in the response to trastuzumab therapy.

**Table 2:** Patients characteristics and treatment response by *PIK3CA* mutation, copy number variation, PTEN, pAkt, and INPP4B expression.

	All patients	*PIK3CA* mutation			*PIK3CA* copy number			PTEN			pAkt			INPP4B		
		WT	Mutation^a^	*P*	Normal/loss	Gain^b^	*P*	Low	High	*P*	Low	High	*P*	Low	High	*P*
N	42	36	7		37	6		12	29		10	30		7	32	
Mean age	53.0	53.1	52.6	0.974	53.6	49.8	0.352	51.8	53.2	0.456	54.9	52.4	0.650	53.6	53.0	0.927
Mean tumor size (mm)+SD	34+16	34+17	32+12	0.843	34+16	30+19	0.344	29+13	35+18	0.295	32+16	35+17	0.839	50+24	30+13	0.035
Nodal status																
negative	21 (49%)	19 (53%)	2 (29%)		18 (49%)	3 (50%)		6 (50%)	14 (48%)		5 (50%)	15 (50%)		3 (43%)	16 (50%)	
positive	22 (51%)	17 (47%)	5 (71%)	0.412	18 (51%)	3 (50%)	0.645	6 (50%)	15 (52%)	1.00	5 (50%)	15 (50%)	1.00	4 (57%)	16 (50%)	1.00
Nuclear grade																
1–2	24 (57%)	21 (60%)	3 (43%)		22 (59%)	2 (40%)		7 (58%)	15 (54%)		6 (67%)	16 (53%)		1 (14%)	20 (65%)	
3	3 (18%)	14 (40%)	4 (57%)	0.438	15 (41%)	3 (60%)	0.636	5 (42%)	13 (46%)	1.00	3 (33%)	14 (47%)	0.704	6 (86%)	11 (35%)	0.031
ER																
negative	23 (53%)	20 (56%)	3 (43%)		19 (51%)	4 (67%)		6 (59%)	16 (55%)		8 (80%)	14 (47%)		3 (43%)	18 (56%)	
positive	20 (47%)	16 (44%)	4 (57%)	0.687	18 (49%)	2 (33%)	0.669	6 (50%)	13 (45%)	1.00	2 (20%)	16 (53%)	0.069	4 (57%)	14 (44%)	0.682
PR																
negative	32 (74%)	29 (81%)	4 (57%)		28 (76%)	5 (83%)		8 (67%)	23 (79%)		10 (100%)	21 (70%)		4 (57%)	25 (78%)	
positive	11 (26%)	7 (19%)	3 (43%)	0.325	9 (24%)	1 (17%)	1.00	4 (33%)	6 (21%)	0.441	0 (0%)	9 (30%)	0.081	3 (43%)	7 (22%)	0.344
Mean ki67+SD	44.7+19	43+19	53+25	0.364	45+20	44+22	0.195	50+22	39+20	0.839	42+22	37+19		42+20	41+18	0.473
Tumor subtype																
luminal/HER2	19 (44%)	15 (79%)	4 (21%)		20 (54%)	4 (67%)		6 (50%)	17 (59%)		8 (80%)	15 (50%)		3 (43%)	19 (59%)	
HER2	24 (56%)	21 (88%)	3 (13%)	0.68	17 (46%)	2 (33%)	0.678	6 (50%)	12 (41%)	0.734	2 (20%)	15 (50%)	0.145	4 (57%)	13 (41%)	0.677
Treatment response																
pCR	26 (60%)	24 (67%)	2 (29%)		22 (59%)	4 (67%)		4 (33%)	21 (72%)		7 (70%)	17 (57%)		3 (43%)	21 (66%)	
non-pCR	17 (40%)	12 (33%)	5 (71%)	0.093	15 (41%)	2 (33%)	1.00	8 (67%)	8 (28%)	0.034	3 (30%)	13 (43%)	0.711	4 (57%)	11 (34%)	0.396

WT; wild type, ER; estrogen receptor, PR; progesteron receptor, HER2; human epidermal growth factor 2, PTEN; phosphatase and tensin homolog, INPP4B; inositol polyphosphate 4-phosphatase-II,

a Mutation was defind as positive if the proportion of mutant DNA was more than 10% by digital PCR analysis.

b copy number gain was defiend if the copy number of *PIK3CA* was greater than 1.5 compared to *RNaseP*.

*PIK3CA* copy number was also analyzed by digital PCR. The median value for the ratio of *PIK3CA* to *RNaseP* was 1.04. In total, 6 cases (14%) showed a ratio>1.5, which was considered as copy number gain. There was no significant difference in pCR rate between the patients with low or normal copy number vs. copy number gain (59% versus 67%, *P* = 1.00).

### Associations between PTEN, pAkt, INPP4B expression and treatment response

**Figure 3:**
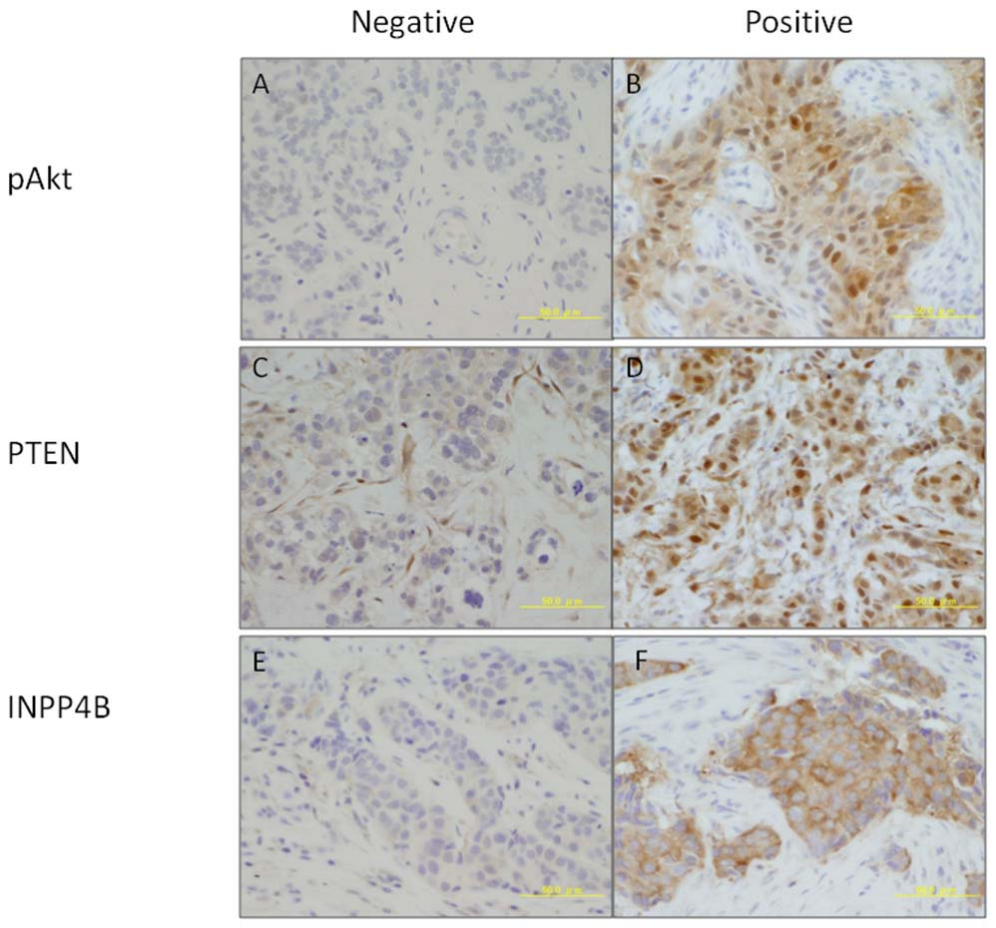
Immunohistochemical staining patterns of PTEN, pAkt, and INPP4B. pAkt expression; A, negative staining (HS<12.5), B, positive (HS≥12.5), PTEN expression; C, negative staining (HS<60), D, positive (HS≥60), INPP4B expression; E, negative staining (HS<60), F, positive (HS≥60) (Magnification ×400).

We then examined the expression of PI3K pathway-related proteins by IHC analyses. Forty-one, 40, and 39 tumors were evaluable for PTEN, pAkt, and INPP4B, respectively. Representative staining patterns for each parameter are shown in Fig. 3. For PTEN and INPP4B, 12 (29%), and 7 (18%) cases, respectively, were considered absent or low expression, and for pAkt 30 cases (75%) were considered high expression. Cases with reduced PTEN expression were less likely to achieve pCR than those with high expression (33% versus 72%, *P* = 0.034, Table 2). We found no significant associations between pAkt, INPP4B and the response.

### Correlations between *PIK3CA* mutation and gene copy number, protein expressions

*PIK3CA* mutation was defined positive if mutant DNA was more than 10% by digital PCR assay for all of the following analyses. Patients with *PIK3CA* mutations tended to have high levels of pAkt expression but the difference was not significant (S3 Table). Three patients (7%) showed both *PIK3CA* mutation and low PTEN expression, and 2 (5%) had *PIK3CA* mutation and low INPP4B. None of the patients had *PIK3CA* mutation and concurrent gain of gene copy number.

### Relationship of each parameter with clinicopathological features

There were no significant correlations of clinicopathological features with *PIK3CA* mutation, gene copy number status, PTEN, or pAkt expression (Table 2). Low INPP4B expression was associated with larger tumor size (*P* = 0.035) and higher nuclear grade (*P* = 0.031), compared to high expression. There were no correlations between, pAKT, PTEN, and INPP4B (data not shown).

### Univariate and multivariate analyses of predictive pCR by biomarker status

Finally, we evaluated the contribution of clinical variables and biomarkers related to the PI3K pathway to the prediction of pCR by logistic regression models (Table 3). Negative of ER and PR status showed higher predictive value in univariate analysis (OR = 0.19; 95%CI 0.05–0.70, and 0.09; 0.02–0.53, respectively). As there was correlation between these two parameters (Spearman coefficient γ = 0.75, *P*<0.001), we included only ER status in the following multivariate analysis, showing its significance remained (OR = 0.14; 0.02–0.78). PTEN expression was also associated with increased chance of pCR in univariate analysis (OR = 5.25; 1.23–22.4), but lost its significance in multivariate analysis. We next defined PI3K pathway activation using patterns of each parameter, meaning *PIK3CA* mutation and expression of PTEN and INPP4B. Tumors with either *PIK3CA* mutations or low PTEN expression were considered to have high PI3K pathway activation, which gave an OR for predictive pCR of 0.11 (95%CI; 0.03–0.48) in univariate analysis, and 0.05 (0.003–0.89) in multivariate analysis. Similarly, if high activation was defined as *PIK3CA* mutation or low expression of PTEN or INPP4B, it was associated with low pCR (OR = 0.15; 0.04–0.63) in univariate analysis, although the significance was borderline in multivariate analysis (OR = 0.14; 0.02–1.12). Thus, activation of PI3K pathway was suggested to be associated with lower response to trastuzumab.

**Table 3:** Logistic regression analyses for predictive pCR.

	Univariate		Multivariate	
	OR (95% CI)	*P*	OR (95% CI)	*P*
Age (year)				
≥50 versus <50	2.13 (0.61–7.41)	0.237		
Tumor size (mm)				
≥30 versus <30	0.76 (0.22–2.59)	0.664		
Nodal status				
positive versus negative	1.31 (0.39–4.47)	0.664		
ER status				
positive versus negative	0.19 (0.05–0.70)	0.013	0.14 (0.02–0.78)	0.025
PR status				
positive versus negative	0.09 (0.02–0.53)	0.007		
Ki67 labeling index (%)				
≥30 versus <30	1.75 (0.42–7.30)	0.442		
Nuclear grade				
3 versus 1, 2	1.12 (0.32–3.91)	0.856		
Tumor subtype				
luminal/HER2 versus HER2	0.39 (0.20–0.77)	0.007		
*PIK3CA* mutational status				
mutation versus wild type	0.20 (0.03–1.19)	0.076		
PTEN expression				
high versus low	5.25 (1.23–22.4)	0.025	0.62 (0.04–9.94)	0.734
INPP4B				
high versus low	2.55 (0.48–13.5)	0.271		
pAkt expression				
high versus low	0.56 (0.12–2.60)	0.459		
*PIK3CA* copy number status				
gain versus normal/loss	1.36 (0.22–8.41)	0.738		
PI3K pathway activation				
ahigh versus normal	0.11 (0.03–0.48)	0.003	0.05 (0.003–0.89)	0.041^d^
bhigh versus normal	0.32 (0.07–1.41)	0.134		
chigh versus normal	0.15 (0.04–0.63)	0.01	0.14 (0.02–1.12)	0.064^e^

OR; odds ratio, CI; confidence interval, ER; estrogen receptor, PR; progesteron receptor, HER2; human epidermal growth factor 2, PTEN; phosphatase and tensin homolog, INPP4B; inositol polyphosphate 4-phosphatase-II.

a high defined as *PIK3CA* mutation and/or PTEN low.

b high defined as PTEN low and/or INPP4B low.

c high defined as *PIK3CA* mutation and/or PTEN low and/or INPP4B low.

d,e The value was indicated when each variable was included in the model, together with ER status and PTEN expression.

## Discussion

In recent years, there has been a concerted effort to identify biomarkers of drug response in neoadjuvant treatment. Although a number of studies regarding the *PIK3CA* gene or PI3K pathway in HER2-positive breast cancer have been reported, they mainly focused on the impact on prognosis or response to anti-HER2 therapy in a metastastic disease [7], [13], [14], [15], [21]. In the present study, we sought to evaluate associations between activation of PI3K pathway and efficacy of trastuzumab therapy in neoadjuvant treatment. The rate of pCR was significantly lower among the patients with high activation of PI3K pathway, who had at least one of *PIK3CA* mutation and/or low PTEN expression, than those without either *PIK3C*A mutation or low PTEN. Further, we performed mutational analysis by digital PCR to complement the direct sequencing data, leading to more accurate measurement of the frequency of *PIK3CA* mutation.

Activating mutations and amplification of the *PIK3CA* gene are commonly found in breast cancer, particularly in ER-positive or HER2-positive disease [11], [12]. There are several studies concerning *PIK3CA* mutation, indicating its importance in determining the survival and treatment efficacy [7], [13]. Jensen et al. showed that patients with *PIK3CA* mutation or increased PI3K pathway activity had poor survival despite adjuvant chemotherapy and trastuzumab [13]. Some similar studies [7], [13], [26] but not all [14], [17] have emphasized the prognostic relevance of *PIK3CA* mutation. With regard to the tumor response, a few studies have shown the predictive meaning of *PIK3CA* mutation [27]. A recent study from the German Breast Group showed that the rate of pCR in their neoadjuvant trials was significantly lower among the patients with HER2-positive disease who harbored *PIK3CA* mutations, than among those who did not (17% versus 37%) [28], [29]. Another neoadjuvant trial (NeoALTTO) which compared trastuzumab- and lapatinib-containing regimens revealed that low PTEN or activating mutation in *PIK3CA* conferred resistance to the trastuzumab regimen, whereas low PTEN was predictive for response to lapatinib [27]. Our study provides additional support for these findings, and further suggests that integrated biomarkers of *PIK3CA* mutation and low PTEN are even stronger predictor of trastuzumab response than either one alone (Table 3), apparently reflecting their interacting roles in the biology of HER2-positive breast cancer. Several ongoing trials targeting the PI3K pathway performed biomarker analysis [30], [31], [32] and found that patients with *PIK3CA* mutations or PTEN loss having a poorer response to trastuzumab therapy may derive increased benefit from PI3K pathway-targeted drugs. Hence, it is of critical importance to understand the mechanisms of trastuzumab resistance and identify those patients before initiation of treatment.

In the present study, we compared two methods of mutational analyses: direct sequencing and digital PCR. The latter is an attractive novel technology that has previously been used for various applications, including mutation detection and analysis of copy number variation [33], [34]. In our study, the digital PCR assay was an extremely useful complement when direct sequencing was unable to identify a mutation. The frequency of patients with mutant DNA found by digital PCR was more than twice that found using direct sequencing (49% versus 21%, Table 1), and appeared to exceed the previously reported frequency (∼39%) [12]. This is presumably due to digital PCR's greater sensitivity in detecting mutant DNA of low abundance, even less than 1%. However, careful consideration is required of the degree to which a small amount of mutation can be implicated in molecular function. In addition, the proportion of mutant DNA detected may depend on the abundance or variation of tumor cells among total genetic content because we used core-biopsy samples for analysis. Due to this issue, we set a cutoff value of the proportion of mutant DNA and found that the pCR rate decreased with elevated proportions of mutant DNA (Fig. 2). To our knowledge, our study is the first to suggest the dose-dependent association of *PIK3CA* mutation with treatment response, though our sample size was limited and further validation in a large population is needed.

Copy number gain of the *PIK3CA* appears to be less common than mutation, having been indentified in 1–14% of breast cancers [35], [36], [37]. A recent study by Gonzalez-Angulo et al. demonstrated that a high copy number of *MET* or *PIK3CA* was associated with poorer prognostic features [38]. No study has reported an association between *PIK3CA* copy number gain and trastuzumab response. Our present study observed no relationship among them, suggesting that a gain of *PIK3CA* gene copy number is not the main molecular event in activating the PI3K pathway in HER2-positive breast cancer.

We also evaluated the expression of the PI3K-pathway-related proteins, pAkt, PTEN, and INPP4B, by IHC. PTEN and INPP4B are negative regulators of the PI3K pathway and are frequently lost in breast cancer [12]. PTEN is a dual phosphatase that mainly dephosphorylates phosphatidylinositol-3,4,5 trisphosphate (PI(3,4,5)P3) to produce PI (4,5)P2 [39]. INPP4B is also a phosphatase which dephosphorylates the 4-position phosphate from PI (3,4)P2 to form PI(3)P [40]. Loss of PTEN or INPP4B results in prolonged activation of Akt and subsequently in increased cell proliferation, migration, and invasion. Consistent with these molecular findings, reduced PTEN expression was associated with lower response to treastuzumab in our study (Table 2). Further, PTEN appeared to be a more robust indicator of pCR following treatment than *PIK3CA* mutation, also in agreement with some previous data [17], [21]. Nagata et al. reported that PTEN activation directly contributes to the antiproliferative effect of trastuzumab, based on the findings that trastuzumab increased PTEN membrane localization and phosphatase activity, leading to decreased PI3K pathway activation and inhibition of proliferation in an *in vitro* and *in vivo* study [8]. For INPP4B expression, we found no the significant impact on the response, although high expression was associated with worse prognostic factors, such as large tumor size and high nuclear grade (Table 2). Recent studies have proved evidence about INPP4B in several human cancers, including prostate cancer [41], melanoma [42], and breast cancer [10], [43]. According to the latter reports, reduced INPP4B has been observed predominantly in basal-like breast carcinoma and appears to be associated with unfavorable patient outcome [10], [43]. Further evidence for any potential role of INPP4B as a biomarker in breast cancer should be accumulated.

In conclusion, we showed that activation of the PI3K pathway as judged by the presence of oncogenic *PIK3CA* mutations or low PTEN expression was associated with a lower frequency of pCR in neoadjuvant treatment. Additionally, we found that digital PCR leads to detection of additional mutations not found by conventional sequencing, and a more accurate determination of the prevalence of *PIK3CA* mutations, thus identifying more patients who are candidates for targeted therapies. In the near future, biomarker analysis to identify women most likely to benefit from the trastuzumab therapy may be conducted routinely in a clinical practice. The present study, thus offers useful evidence that the PI3K-pathway-related components *PIK3CA*, PTEN and INPP4B may be candidate predictive biomarkers for trastuzumab therapy.

## Materials and Methods

### Subjects and tissues

A total of 43 patients with HER2-positive breast cancer who received both neoadjuvant treatment and surgery at Kumamoto University Hospital between 2004 and 2012 were selected. All patients underwent pretreatment biopsies using a needle with a more than 14G needle and were diagnosed with invasive breast carcinoma. We evaluated biomarkers in the pretreatment specimens. Neoadjuvant chemotherapy and trastuzumab treatment was assigned to each patient according to their risk on the basis of clinical parameters, and in accordance with the recommendation of the St. Gallen International Expert Consensus on primary therapy of early breast cancer at the time. Only patients who received at least six courses of treatment (commonly up to eight courses) with anthracycline or taxane-containing regimens in combination with trastuzumab were included. The representative regimens of chemotherapy were as follows; FEC (5-fluorouracil 500 mg/m^2^, epirubicin 100 mg/m^2^, and cyclophosphamide 500 mg/m^2^, every 3 weeks) followed by docetaxel (75 mg/m^2^, every 3 weeks) or paclitaxel (80 mg/m^2^, every week) each for 4 cycles, TC (docetaxel 75 mg/m^2^ and cyclophosphamide 600 mg/m^2^, every 3 weeks) for 6 cycles.

### Ethics Statement

Written informed consent was obtained from all subjects for the collection and research use of breast tumors. Our complete study was approved by the ethics committee of Kumamoto University Graduate School of Medical Sciences.

### Evaluation of treatment response

The response of primary breast cancer during treatment was evaluated using clinical diagnostic imaging (ultrasound and magnetic resonance imaging). The achievement of pCR on postoperative specimens was defined as the absence of invasive residuals in breast or nodes. Noninvasive breast residuals were allowed.

### Immunohistochemical analysis

Histological sections (4 µm) were deparaffinized and incubated for 10 min in methanol containing 0.3% hydrogen peroxide. They were then immunostained with monoclonal antibodies against ERα (SP1; Ventana Japan, Tokyo, Japan), progesterone receptor (PR) (1E2; Ventana Japan), HER2 (4B5; Roche, Tokyo, Japan), Ki67 (MIB1; Dako Japan, Kyoto, Japan), PTEN (136G6; 1∶200, Cell Signaling Technology, Tokyo, Japan), pAkt (Ser473) (D9E; 1∶50, Cell Signaling Technology) and INPP4B (EPR3108; 1∶100, Abcam). The staining was carried out in a NexES IHC immunostainer (Ventana Medical Systems, Tucson, AZ), in accordance with the manufacturer's instructions. ER and PR were considered positive if more than 1% of nuclei were stained. HER2 expression was also determined by IHC staining based on the Hercep test. We considered a tumor to be HER2-positive if the specimen either scored 3+ by IHC, or showed HER2/CEP17 ratio with≥2.2 in fluorescence *in situ* hybridization (FISH) according to the 2007 ASCO/CAP guideline [44]. Tumor subtypes were defined according to the expression of ER, PR and HER2. Ki67 was scored as the percentage of nuclear-stained cells out of all cancer cells in the hot spot of the tumor regardless of the intensity in a ×400 high-power field (Ki67 labeling index [45]). We counted between 500 and 1,000 tumor cells as recommended by the International Ki67 in Breast Cancer Working Group [46]. For PTEN, pAkt and INPP4B expression, the H-score was calculated by multiplying the percentage of positive cells (0–100) by the staining intensity score (0–3). PTEN expression level was scored semiquantitatively based on the immunoreactive score (IRS) as described by Sakr RA et al. [47]. Low PTEN expression was considered as H-score <60. Similarly, the H-score cutoffs for INPP4B and pAkt were set at 60 and 12.5, respectively.

### Mutational analysis

Genome DNA from formalin-fixed paraffin-embedded (FFPE) tissue samples which included more than 3 tissue cores was isolated using the AllPrep DNA/RNA Mini kit (Qiagen, Germantown, MD, USA) according to the manufacturer's instructions. The extracted DNA was quantified using a Nanodrop spectrophotometer (Thermo Scientific, Tokyo, Japan).

#### Direct sequencing analysis

First, all samples were genotyped by direct dideoxynucleotide sequencing. PCR primers used for amplifying segments were designed for analysis of *PIK3CA* hotspot mutations in exon 9 and exon 20. These sequences are shown in S1 Table. Each PCR was performed on 50 ng of genomic DNA with AmpliTaq Gold 360 Master Mix (Applied Biosystems, CA, USA) according to the manufacturer's protocol. PCR products were purified using a QIAquick PCR Purification Kit (Qiagen) and were sequenced using the BigDye Terminator v3.1 Cycle Sequencing Kit (Life Technologies, Tokyo, Japan) and an ABI 3130 automated capillary sequencer. We used 6 ng of PCR product as a DNA template in sequencing reactions.

#### Analysis by digital PCR

We used Custom TaqMan SNP Genotyping assays (Applied Biosystems), consisting of a pair of primers and two TaqMan probes, for the detection of 3 common mutations in *PIK3CA* (E542K, E545K, and H1047). One probe was specific to the wild-type sequence and another was specific to the variants of corresponding mutations. The sequences of the primers and TaqMan MGB probes are described in S1 Table. Digital PCR was performed using the BioMark System with the BioMark qdPCR37K IFC (Fluidigm, Tokyo, Japan) according to the manufacturer's instructions. We prepared 4-*µ*L reaction mixes containing 2 *µ*L of TaqMan Gene expression Master Mix (Applied Biosystems), 0.4 *µ*L of 20×GE sample loading reagent, 0.2 *µ*L of 20×gene-specific assay, 0.2 *µ*L of DNA-free water, and 1.2 *µ*L of target gDNA (24 ng). PCR runs were as follows; 120 s at 50°C, a hot start at 95°C for 10 min, and 40 cycles of 15 s of denaturation at 95°C and 1 min of annealing and extension at 60°C. The number of positive amplified signals in each panel was used for quantifying the different DNA sequences and analysis of PCR data was done using the BioMark Digital Array software. This uses a Poisson model to estimate the number of DNA molecules from the count of positive wells. Finally, the proportion of mutant signals in total signals was regarded as a mutant allele frequency.

### *PIK3CA* copy number assay

For the copy number application, we used the BioMark System with the BioMark qdPCR37K IFC (Fluidigm) as described above. *RNase P*, which is regarded as a reference for gene dosage, was substituted for 0.2 *µ*L of DNA-free water in the 4-*µ*L reaction mixes. The reactions were performed in the panels including both *PIK3CA* and *RNaseP* assays. Each probe was obtained for TaqMan copy number assay (4316844 for *RNase P,* Hs02708380_cn, for *PIK3CA*, Applied Biosystems). We count the number of positive chambers for each and estimated *PIK3CA*: *RNase P* ratio as a relative copy number.

### Statistical analysis

The significance of differences in categorized demographic variables was evaluated using Chi-square or Fisher's exact test and the nonparametric Mann-Whitney *U* test. Logistic regression methods were adopted for univariate and multivariate analyses to assess the associations of clinical and biological parameters with pCR. Odds ratios (ORs) and 95% confidence intervals (CIs) were calculated. All statistical analyses were carried out using STATA ver.12 (Stata Corp, College Station, TX). All tests were two-sided and *P* values <0.05 were considered statistically significant.

## Supporting Information

S1 Table **Oligonucleotide sequences for sequencing and digital PCR.**
(XLSX)

S2 Table **Patient characteristics before trastuzumab therapy.**
(XLSX)

S3 Table **Correlations between PIK3CA mutation and biomarker status.**
(XLSX)
